# Heterodimeric IL-15 delays tumor growth and promotes intratumoral CTL and dendritic cell accumulation by a cytokine network involving XCL1, IFN-γ, CXCL9 and CXCL10

**DOI:** 10.1136/jitc-2020-000599

**Published:** 2020-05-26

**Authors:** Cristina Bergamaschi, Hrishikesh Pandit, Bethany A Nagy, Dimitris Stellas, Shawn M Jensen, Jenifer Bear, Maggie Cam, Antonio Valentin, Bernard A Fox, Barbara K Felber, George N Pavlakis

**Affiliations:** 1 Human Retrovirus Pathogenesis Section, Vaccine Branch, Center for Cancer Research, National Cancer Institute at Frederick, Frederick, Maryland, USA; 2 Human Retrovirus Section, Vaccine Branch, Center for Cancer Research, National Cancer Institute at Frederick, Frederick, Maryland, USA; 3 Robert W Franz Cancer Research Center, Providence Portland Medical Center, Earle A Chiles Research Institute, Portland, Oregon, USA; 4 Office of Science and Technology Resources, Center for Cancer Research, National Cancer Institute, Frederick, Maryland, USA

**Keywords:** immunotherapy, dendritic cells, cytokines, CD8-positive T-lymphocytes, T-lymphocytes

## Abstract

**Background:**

Interleukin-15 (IL-15) promotes growth and activation of cytotoxic CD8^+^ T and natural killer (NK) cells. Bioactive IL-15 is produced in the body as a heterodimeric cytokine, comprising the IL-15 and IL-15 receptor alpha chains (hetIL-15). Several preclinical models support the antitumor activity of hetIL-15 promoting its application in clinical trials.

**Methods:**

The antitumor activity of hetIL-15 produced from mammalian cells was tested in mouse tumor models (MC38 colon carcinoma and TC-1 epithelial carcinoma). The functional diversity of the immune infiltrate and the cytokine/chemokine network within the tumor was evaluated by flow cytometry, multicolor immunohistochemistry (IHC), gene expression profiling by Nanostring Technologies, and protein analysis by electrochemiluminescence and ELISA assays.

**Results:**

hetIL-15 treatment resulted in delayed primary tumor growth. Increased NK and CD8^+^ T cell tumoral infiltration with an increased CD8^+^/Treg ratio were found by flow cytometry and IHC in hetIL-15 treated animals. Intratumoral NK and CD8^+^ T cells showed activation features with enhanced interferon-γ (IFN-γ) production, proliferation (Ki67^+^), cytotoxic potential (Granzyme B^+^) and expression of the survival factor Bcl-2. Transcriptomics and proteomics analyses revealed complex effects on the tumor microenvironment triggered by hetIL-15 therapy, including increased levels of IFN-γ and XCL1 with intratumoral accumulation of XCR1^+^IRF8^+^CD103^+^ conventional type 1 dendritic cells (cDC1). Concomitantly, the production of the chemokines CXCL9 and CXCL10 by tumor-localized myeloid cells, including cDC1, was boosted by hetIL-15 in an IFN-γ-dependent manner. An increased frequency of circulating CXCR3^+^ NK and CD8^+^ T cells was found, suggesting their ability to migrate toward the tumors following the CXCL9 and CXCL10 chemokine gradient.

**Conclusions:**

Our results show that hetIL-15 administration enhances T cell entry into tumors, increasing the success rate of immunotherapy interventions. Our study further supports the incorporation of hetIL-15 in tumor immunotherapy approaches to promote the development of antitumor responses by favoring effector over regulatory cells and by promoting lymphocyte and DC localization into tumors through the modification of the tumor chemokine and cytokine milieu.

## Background

Immunotherapy has emerged as a valuable strategy for the treatment of several type of cancers.^1 2^ The clinical use of cytokines, antibodies targeting checkpoint inhibitors and adoptive cell transfer is increasing, although only a subset of patients benefits from these approaches. The presence of tumor-infiltrating effector T cells is considered a favorable prognostic indicator in many cancer types.^3^ In colorectal cancer, the infiltration of cytotoxic memory T cells within the tumor center and invasive margin represents an important predictor of survival.^4–6^ During programmed cell death protein-1 blockade treatment, higher numbers of CD8^+^ T cells are found within the tumor of responder patients.^7^ Several studies suggest that the ratio of CD8^+^/Treg cells within the tumor is an important predictor of cancer immunotherapy success.^8^ The presence of intratumoral conventional type 1 dendritic cells (cDC1) has also been positively associated with patient survival in multiple cancers.^9 10^ In preclinical models, abundance of cDC1 within the tumor microenvironment is associated with tumor regression and considered critical for the success of T cell-based immunotherapies.^11–13^ cDC1 development requires expression of the transcription factors Interferon regulatory factor 8 (IRF8) and Basic leucin zipper transcriptional factor ATF-like 3 (Batf3)^14^; they can be identified by surface expression of CD11c, the X-C Motif Chemokine Receptor 1 (XCR1)^15 16^ and the C-type lectin receptor DNGR-1.^17^ In non-lymphoid tissues, including tumors, cDC1 cells also express integrinαE (CD103), whereas expression of CD11b is low.^10 12 18^ The recruitment and retention of cDC1s within the tumor are regulated by chemokines released by cancer cells, stroma and intratumoral lymphocytes.^11 19^ The antitumoral activity of cDC1 relates to their ability to cross-present tumor antigens taken up within the tumor microenvironment, and to initiate primary T cell responses in the tumor-draining lymph nodes,^12 18 20^ enhancing local cytotoxic lymphocyte function.^10 21^ Additionally, cDC1 may contribute to the recruitment of effector T cells by releasing Chemokine (C-X-C motif) ligand 9 (CXCL9) and Chemokine (C-X-C motif) ligand 10 (CXCL10),^13 22 23^ establishing a favorable chemokine gradient within the tumor. Analyses of the tumor cytokine landscape have shown that interferon-γ (IFN-γ)^24^ and interleukin-15 (IL-15)^25–27^ signatures are also associated with beneficial antitumor responses.

IL-15 is of great interest to the cancer immunotherapy field due to its ability to promote survival and stimulate several leucocyte subsets including natural killer (NK), CD8^+^ and γδ T cells^28^ that possess antitumor properties. In phase I clinical trials, *Escherichia coli*-derived single-chain IL-15 was shown to expand NK and CD8^+^ T cells, but the short plasma half-life^29^ and immunogenicity in humans^29 30^ may limit the therapeutic use of this molecule. IL-15 expression in vivo requires the intracellular binding of IL-15 to IL-15 receptor α (IL-15Rα) in producer cells.^31–34^ Indeed, we have reported that bioactive IL-15 in vivo comprises a complex of the IL-15 chain with the IL-15Rα chain, which together form the IL-15 heterodimer (named hetIL-15).^35 36^ The two chains, IL-15 and IL-15Rα, are produced coordinately in the same cell and associate rapidly in the endoplasmic reticulum^36 37^ due to their high binding affinity (k_d_ ~10^-11^). hetIL-15 is then transported to the cell surface and, on proteolytic cleavage of IL-15Rα, released as bioactive soluble heterodimeric molecule.^35 36 38–40^ hetIL-15 produced from human HEK293 cells is well tolerated and bioactive in both mice^41^ and rhesus macaques.^42 43^ Therapeutic efficacy of hetIL-15 and molecules composed of parts of IL-15 and IL-15Rα has been shown in preclinical cancer models,^40 44–48^ and several molecules similar to hetIL-15 are currently being evaluated in cancer immunotherapy clinical trials (NCT02452268 and^49 50^). We have shown in the B16 melanoma model that hetIL-15 enhances the survival, proliferation and antitumor effects of adoptively transferred Pmel-1 cells without the need for lymphodepletion preconditioning, resulting in improved tumor control and survival.^47^ Overall, hetIL-15 administration is associated with increased proliferation of effector memory T lymphocytes and NK cells expressing the cytotoxic serine protease Granzyme B (GzmB).^47^ However, the mechanism(s) underlying promotion of antitumor responses by IL-15 is still not completely understood.

The objectives of this study were to explore how hetIL-15 promotes lymphocyte entry into the tumor and to characterize the interactions between tumor-infiltrating lymphocytes (TILs) and other cell types through the analysis of cytokines and chemokines affected by hetIL-15 therapy.

## Methods

### Mice

C57BL/6 mice were obtained from Charles River Laboratory and Envigo (Frederick, MD). IL-15 knock out (KO) mice were purchased from Taconic. IFN-γ KO were kindly provided by the Cancer Inflammation Program, National Cancer Institute.

### Mouse tumor models and hetIL-15 immunotherapy

Murine carcinoma-38 (MC38) colon carcinoma and Tissue culture-1 (TC-1) lung epithelial cell-derived carcinoma cells were maintained in Dulbecco's Modified Eagle Medium (DMEM) supplemented with 10% heat-inactivated fetal bovine serum (Sigma), penicillin/streptomycin, essential amino acids and 4-(2-hydroxyethyl)-1-piperazineethanesulfonic acid (HEPES). Animals aged 6–8 weeks received 1–2×10^5^ tumor cells by subcutaneous (SC) injection in the flank. Five days after inoculation of tumor cells, tumor-bearing mice were randomized into two groups, untreated and hetIL-15 treated. hetIL-15 was purified from HEK293 cells (Admune Therapeutic LLC/Novartis). For hetIL-15 treatment, mice received intraperitoneal (IP) injection of 3 µg (molar mass of IL-15) of hetIL-15^41 51^ three times/week for a total of 4–8 injections. Tumor area (length x width) was measured every 2–3 days in a blinded fashion. To block protein secretion from tumor cells in vivo, 100 µg brefeldin (Sigma) at a concentration of 10 mg/mL were delivered intratumorally (IT), 30 min before sacrifice and tumor excision, in some experiments.

### Preparation of single cell suspension and flow cytometry

Excised tumors were cut into small pieces and digested using the Tumor Dissociation Kit (130-096-730, Miltenyi Biotec), as per manufacturer’s instructions. In some experiments, CD8^+^ T cells were isolated from tumor cell suspensions using Dynabeads FlowComp Mouse CD8 Kit (ThermoFisher Scientific), as per manufacturer’s instructions. Spleens were dissociated using a 100 µm cell strainer and washed to remove any remaining organ stroma. Cells were washed and stained with a fixable viability dye (ThermoFisher Scientific) for 30 min at 4°C. Surface staining was performed using antibodies for the following markers: CD3 (145–2 C11), CD4 (RM4-5), CD8 (53–6.7), CD45 (30-F11), CD49b (DX5), CD64 (X54-5/7.1), CD19 (ID3) from BD Biosciences; CD11c (N418), CD11b (M1/70), MHC-II (M114.15.2), XCR-1 (ZET), CXCR3 (CXCR3-173) from Biolegend and CD103 (2E7) from ThermoFisher Scientific. For intracellular staining, cells were fixed and permeabilized using the Foxp3 staining buffer (ThermoFisher Scientific), following manufacturer’s instructions. Samples were stained with Ki67 (SOLA15) and B-cell lymphoma-2 (Bcl-2) (3F11) from BD Biosciences; Foxp3 (FJK-16s) and IRF8 (V3GYWCH) from ThermoFisher Scientific; GzmB (GB11) and CXCL9 (MIG-2F5.5) from Biolegend; and X-C Motif Chemokine Ligand 1 (XCL1) (BAF486) from R&D Systems followed by PE-Streptavidin (BD Biosciences). Cells were acquired in a Fortessa flow cytometer (BD Biosciences). Data analysis was performed using FlowJo software (Tree Star, Ashland, Oregon, USA). Analyses of tumor immune infiltrates were performed in two to three independent experiments for each type of tumor.

### In vitro stimulation of TILs

Tumor cell suspensions were seeded in 24-well plates in the presence of beads conjugated with α-CD3/CD28 antibodies (Dynabeads Mouse T Activator CD3/CD28, 11 452D, ThermoFisher Scientific) and incubated at 37°C in the presence of anti-CD107a antibody (1D4B, BD Biosciences) and monensin (GolgiStop, BD Biosciences). Similar cultures without antibody-stimulation were used as negative controls. After 6 hours of incubation, cells were harvested and stained for surface markers and intracellular IFN-γ (XMG1.2, BD Biosciences).

### Immunohistochemistry

Immunohistochemistry (IHC) was performed as previously reported.^52^ After deparaffinization, 4.5 µm thick sections were incubated with rabbit anti-CD3 (SP7, Abcam, ab16669, 1:100), followed by treatment with tyramide-fluorophore reagent (PerkinElmer, NEL791001KT; Life Technologies, T20950) at 1:100 dilution in Amplification plus buffer (PerkinElmer, NEL791001KT) for 10 min at room temperature (RT) and washed in Tris-Buffered Saline, 0.1% Tween^®^20 Detergent (TBS-T) and H_2_0. Similar procedure was performed for the rat anti-CD8 (4SM15, ThermoFisher, 14-0808-82, 1:2000), rat anti-CD4 (4SM95, ThermoFisher, 14–9766, 1:200) and rat anti-Foxp3 antibodies (FJK-16s, ThermoFisher, 145 773–82, 1:100) using an anti-rat secondary HRP (Vector Labs, MP-7444–15). After washes in TBS-T, 4′,6-diamidino-2-phenylindole (DAPI) (Life Technologies, D1306, 1 mg/mL stock, 1:500 in Phosphate buffered saline (PBS)) was added to slides for 10 min at RT. Slides were imaged at both 4 x, and 20 x using Vectra imaging software (PerkinElmer), and the number of cells were enumerated from the top nine hotspots images, to provide equal weighting for each mouse, using inForm analysis software (PerkinElmer).

### Analysis of cytokine and chemokine levels in tumor lysates

Excised tumors were resuspended in T-Per Tissue Protein Extraction Reagent (ThermoFisher Scientific) and homogenized using 2.8 mm ceramic (zirconium oxide) beads (Precellys Lysing kit) and Precellys Evolution Homogenizer. Tumor lysates were recovered after spin at 13 000 rpm for 15 min at 4°C and analyzed for cytokine levels using U-PLEX Biomarker group 1 mouse Assay (35 analytes; Meso Scale Diagnostics) and ELISAs specific for the mouse chemokines XCL1 (ThermoFisher Scientific) and CXCL9 (ThermoFisher Scientific), following manufacturers instructions.

### Gene expression analysis by nCounter PanCancer immune profiling panel

Tumors were mechanically disrupted in RLT buffer (QIAGEN) using 2.8 mm ceramic beads (Precellys Lysing kit) and Precellys Evolution Homogenizer. RNA extraction was performed with RNeasy (QIAGEN) including on-column DNase I digestion, according to the manufacturer’s instructions. nCounter PanCancer Immune Profiling Panel (NanoString Technologies) was used to monitor the expression of a panel of 780 genes related to immuno-oncology. The mRNA molecules were counted with the NanoString nCounter at the Laboratory of Molecular Technology (Advanced Technology Program, Frederick National Laboratory). Analysis was performed with a workflow written in R and through a user interface developed on the Foundry Platform (Palantir Technologies). Raw data were filtered to remove low expressed genes, leaving 758 genes out of the original 780. To normalize data, log transformation and quantile normalization were applied. To define differentially expressed genes, we run limma^53^ and set thresholds at two-fold change and p<0.05 difference between groups (adjusted p value following Bonferroni multiple comparison testing). Gene enrichment analysis on the Gene Ontology (GO) database was subsequently performed on the top 150 genes (sorted by t-statistic) using the Fisher’s exact test (https://github.com/CCBR/l2p). Heatmaps were represented as Z-score centered and rescaled. To determine immune cell scores, the geometric mean of the cell marker genes were calculated from normalized gene expression values within each sample. Cell type gene markers for cytotoxic cells and DCs were determined as in Danaher *et al.* 54

### Statistics

Differences between untreated and hetIL-15 treated mice were evaluated by a two-tailed Mann-Whitney U test. Tumor areas were plotted as mean±standard error of the mean (SEM) for each data point, and tumor growth curves were compared using mixed-effects analysis of variance (ANOVA). The p values were corrected for multiple comparisons using Holm-Sidak test. Prism V.8.1 software package was used for analysis.

## Results

### hetIL-15 induces intratumoral accumulation of CD8+ T and NK cells resulting in control of tumor growth

**Figure 1:**
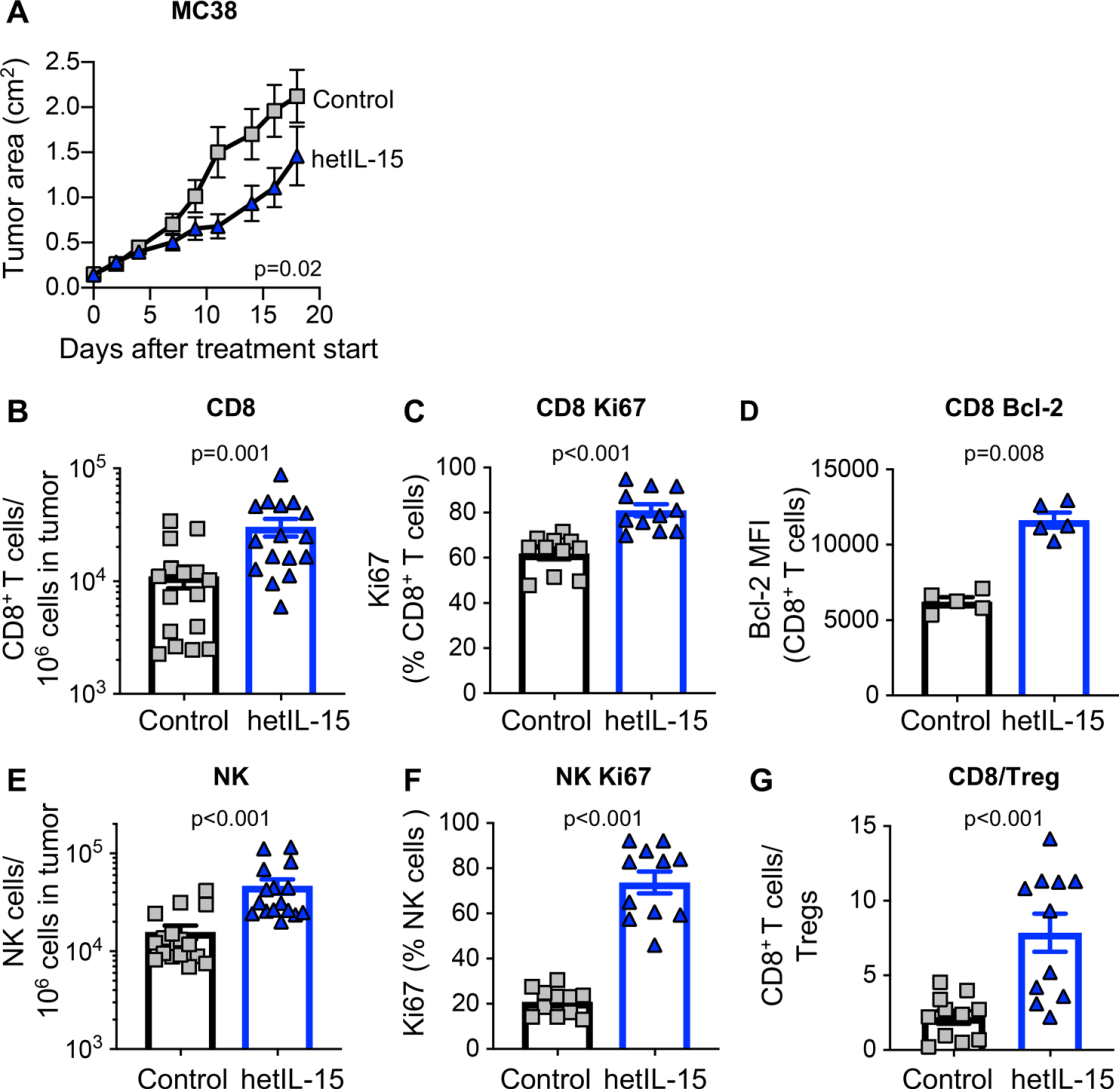
hetIL-15 delays tumor growth and promotes tumor-infiltrating lymphocytes proliferation and survival. Mice, implanted with 2×10^5^ MC38 cells at day −5, were randomized in two different treatment groups: hetIL-15 (blue traingle) and PBS (gray square) administration. hetIL-15 (3 µg/injection/mouse) was injected IP three times per week. (A) Control mice (n=10) and hetIL-15-treated mice (n=9) received eight injections, starting at day 0, and tumor growth was monitored overtime. Tumor size measurements were performed every 2 to 3 days. Tumor area (length×width) mean±SEM for each time point are shown. Similar results were obtained in three independent experiments. (B–G) MC38 tumor-bearing mice were sacrificed at day eight after treatment with either PBS or hetIL-15 (1 day after the fourth administration). Tumor immune infiltrates were analyzed by flow cytometry to determine: (B) frequency of tumor-infiltrating CD8^+^ T cells. Data of three independent experiments were combined; (C) percentage of dividing tumor infiltrating CD8^+^T cells; (D) expression of the survival factor Bcl-2 in the tumor-infiltrating CD8^+^ T cell population; (E) frequency of tumor-infiltrating NK cells; (F) percentage of dividing tumor infiltrating NK cells; (G) intratumoral CD8^+^ T cells/Treg ratio for each treatment group. Bars represent mean±SEM. P values are from Mann-Whitney U test. hetIL-15, heterodimeric interleukin-15; IP, intraperitoneal; MFI, mean fluorescence intensity; NK, natural killer; SEM, Standard error of the mean.

**Figure 2:**
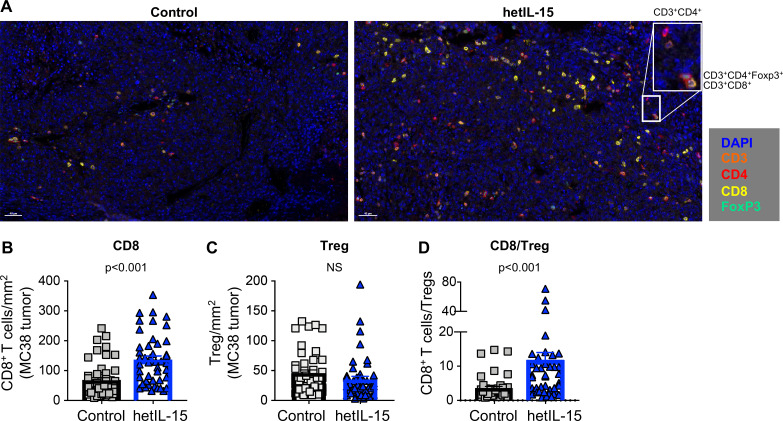
CD8^+^ T cells accumulation and increased CD8^+^/Treg ratio in MC38 tumors from mice treated with hetIL-15. (A) Immunohistochemistry staining of MC38 tumor sections from control (left panel) and hetIL-15-treated mice (right panel). Analysis was performed at day eight after treatment with either PBS or hetIL-15 (1 day after the fourth administration). TILs were monitored with CD3 (orange), CD4^+^ (red), CD8^+^ (yellow) and FOXP3 (green) antibodies. A representative image (×20 magnification) from one mouse/group is shown. Insert represents a ×4 magnification. (B–D) The number of CD8^+^ T cells per mm^2^ (B), the number of Tregs per mm^2^ (C), and the ratio of CD8^+^ T cells/Treg cells (D). Data are from nine ×20 ‘hotspot’ fields (containing the highest number of CD8^+^ T cells/mm^2^) per mouse. Six mice in each treatment group were analyzed. P values are from Mann-Whitney U test. hetIL-15, heterodimeric interleukin-15; TILs, tumor-infiltrating lymphocytes.

To address the role of endogenous IL-15 in tumor growth, MC38 colon carcinoma cells were administered SC to C57BL/6 wild type (wt) and age-matched IL-15 KO mice, and tumor development was monitored overtime. Tumors showed an accelerated growth in mice lacking IL-15 compared with wt mice (online supplementary figure 1), suggesting that IL-15-dependent immune mechanisms can limit the development of tumors. We next used human cell-produced hetIL-15 treatment as an anticancer immunotherapeutic drug in the MC38 colon carcinoma mouse model. hetIL-15 administration, following a 2-week schedule with three injections/week and a dose of 3 µg/injection by IP delivery, resulted in a significant delay of tumor growth (figure 1A). Analysis of the tumor immune infiltrate from untreated and hetIL-15 (day 8, 1 day after the fourth administration) treated mice was performed by both flow cytometry (figure 1B–G) and IHC (figure 2). hetIL-15 treatment increased the number of intratumoral CD8^+^ T cells by ~3 x (figure 1B). The proliferation rate of intratumoral CD8^+^ T cells in untreated mice was ~50%–60%, suggesting rapid lymphocyte turnover in tumor sites. On hetIL-15 treatment, the percentage of Ki67^+^ CD8^+^ T cells significantly increased to ~80% (figure 1C). Tumor-infiltrating CD8^+^ T cells were also characterized by a significant upregulation in the expression of Bcl-2 in comparison to control mice, indicative of increased survival (figure 1D). Similar results were also observed in the NK cells analyzed in MC38-bearing mice (figure 1E–F). hetIL-15 treatment promoted a ~3 x increased accumulation of NK cells in the tumor sites (figure 1E) and these NK cells showed significantly higher proliferation rate in comparison to control mice (figure 1F).

10.1136/jitc-2020-000599.supp1 Supplementary data

Favorable outcome in cancer immunotherapy treatments has also been linked to the ratio of CD8^+^ T cells to Tregs.^8^ For this reason, we determined the CD8^+^/Treg ratio within the tumors from untreated and hetIL-15 treated mice. As previously reported in the B16 melanoma model,^47^ MC38 tumors were characterized by a significant infiltration of Treg cells with a CD8^+^/Treg ratio of ~2. hetIL-15 promoted a significant accumulation of intratumoral CD8^+^ T cells resulting in a ~5 x increase in the CD8^+^/Treg ratio (figure 1G).

The lymphocyte infiltrate in MC38 tumors was also analyzed by IHC (figure 2). Staining of tumor sections with antibodies against CD3, CD4, CD8 and Foxp3 confirmed that the lymphocytes isolated on in vitro digestion were of intratumoral origin rather than peripherally associated with the excised tumors (figure 2A). Quantification of CD8^+^ T cells/mm^2^ (figure 2B) by IHC showed a significant increase in the hetIL-15 group, with lymphocytes distributed throughout the tumor. In contrast, hetIL-15 treatment did not affect the number of tumor-infiltrating Tregs (figure 2C). As a consequence of CD8^+^ T cell expansion, a significant increase in the CD8^+^ T cells/Treg ratio was also observed by IHC on hetIL-15 administration (figure 2D).

The anticancer efficacy of hetIL-15 was verified in the mouse TC-1 carcinoma model using the same methods (online supplementary figure 2A). hetIL-15 treatment was associated with significantly increased intratumoral CD8^+^ T cells, unchanged frequency of tumor infiltrating Tregs and increased CD8^+^/Treg ratio (online supplementary figure 2B–F).

The systemic effects of hetIL-15 treatment were evaluated in MC38-bearing mice. hetIL-15 induced a significant increase in the absolute number of CD8^+^ T and NK cells in spleen (online supplementary figure 3A–D, respectively) with a significant upregulation in the expression of Ki67^+^ cells (online supplementary figure 3B–E, respectively) and the survival marker Bcl-2 (online supplementary figure 3C).

Taken together, these data show that hetIL-15 sustains proliferation and survival of lymphocytes both in tumor and spleen, and verify the efficacy of hetIL-15 as anticancer immunotherapeutic agent.

### hetIL-15 induces T cells with an effector-like gene signature and promotes their cytotoxic functions

**Figure 3:**
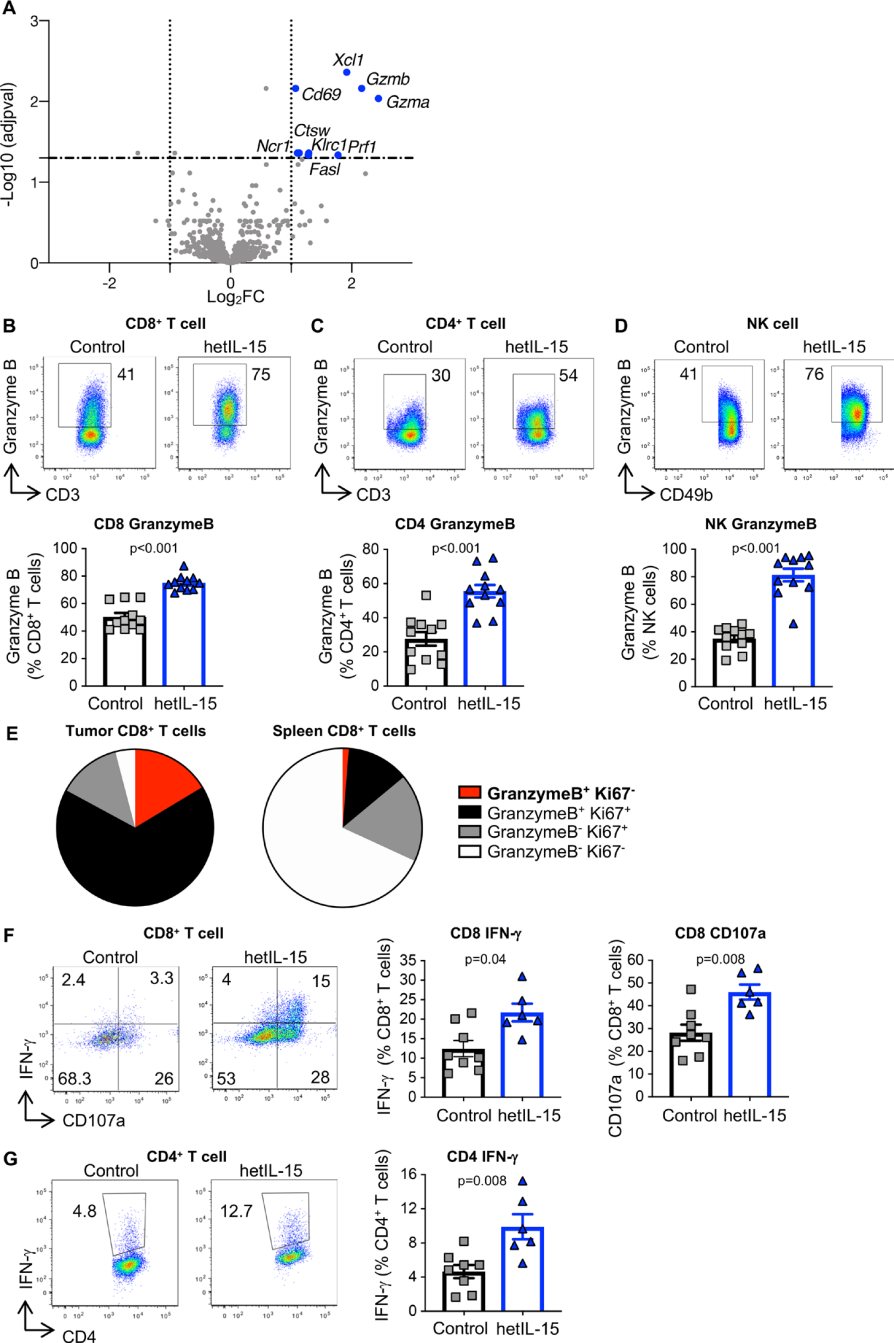
Tumors from hetIL-15-treated mice comprise lymphocytes with an effector-like gene signature and enhanced cytotoxic functions. (A) Gene expression analysis from MC38 tumors recovered from mice treated with either PBS (n=5) or hetIL-15 (n=6) was performed by the Nanostring technology using a panel of 780 immune-oncology related gene probes. The analysis was conducted at 3 hours after the fourth administration. Volcano plot depicts differentially expressed genes between the two treatment groups, highlighting the upregulated genes (blue dots) on hetIL-15 treatment. To define differentially expressed genes, we used one log2 change (vertical dotted lines) and p<0.05 (adjusted p value for multiple comparison; horizontal broken line) difference between groups. (B–D) Tumor-resident CD8^+^ T cells (B), CD4^+^ T cells (C) and NK cells (D) were analyzed for the expression of the cytotoxic marker GzmB by intracellular staining followed by flow cytometry. Dot plots from a representative animal (upper panels) and the percentage of GzmB^+^ cells within each cell subset (bottom panel) are shown. (E) Pie charts show the proportion of GzmB^+^Ki67^-^(red), GzmB^+^Ki67^+^(black), GzmB^-^Ki67^+^(gray) and GzmB^-^Ki67^-^(white) cells within the total CD8^+^ T cell subset in tumor (left panel) and spleen (right panel) of hetIL-15 treated animals. (F–G) IFN-γ production and degranulation (CD107) in tumor-infiltrating CD8^+^ T cells (F) and CD4^+^ T cells (G) on ex vivo stimulation with beads coated with anti-CD3/CD28 antibodies. Dot plots show a representative animal from each group. Bars represent mean±SEM. P values are from Mann-Whitney U test. hetIL-15, heterodimeric interleukin-15; IFN-γ, interferon-γ; NK, natural killer; GzmB, Granzyme B; SEM, Standard error of the mean.

We performed gene expression analysis of MC38 tumors using a panel of 780 immune-oncology related gene probes (Nanostring Technology). Tumors were excised 3 hours after the fourth hetIL-15 administration and processed for mRNA isolation. Comparison of gene expression identified several significantly overexpressed genes in tumors recovered from hetIL-15-treated mice (figure 3A). *Gzmb* and *Gzma* were the most upregulated genes (~5 x, adjusted p<0.01). *Xcl1, Prf1, Ctsw, CD69, Klrc1, Ncr1* and *Fasl* were also significantly overexpressed in hetIL-15-treated mice (figure 3A). These upregulated genes after hetIL-15 treatment represent an expression signature that corresponds to activated TILs with cytotoxic phenotype. Nanostring analysis also identified additional functional pathway signatures, including signal transducer and activator of transcription intracellular signaling, T-cell receptor (TCR) recognition of cognate antigen, IFNs signaling, increased metabolic rate and immune cell chemotaxis (online supplementary figure 4).

To confirm the transcriptomic data, we analyzed the GzmB content of TILs. Flow cytometric analysis demonstrated that provision of hetIL-15 resulted in higher proportion of tumor-infiltrating CD8^+^ and CD4^+^ T lymphocytes, as well as NK cells harboring GzmB in comparison to untreated mice (figure 3B–D, respectively). Thus, hetIL-15 treatment led to a significant accumulation of GzmB^+^ CD8^+^, CD4^+^ T and NK cells per tumor. Similar results were obtained in the TC-1 tumor model (online supplementary figure 2G). We also analyzed the GzmB content of splenic CD8^+^ T and NK cells. In untreated animals, both CD8^+^ T and NK cells were negative for GzmB, but on hetIL-15 administration, we observed a significant increase in the frequency of lymphocytes harboring GzmB (online supplementary figure 3F and G, respectively). Interestingly, we identified an intratumoral CD8^+^ T cell subset characterized by the expression of GzmB and lack of Ki67 (figure 3E, red) in hetIL-15 treated mice, but these cells were almost completely absent within the spleen. These GzmB^+^Ki67^-^ CD8^+^ T cells are indicative of a terminally differentiated cytotoxic phenotype and represented ~15% of the total CD8^+^ T cells within the tumors (figure 3E). These data indicate that hetIL-15 treatment preferentially induces intratumoral accumulation of CD8^+^ T cells committed to killing.

We also investigated the production of IFN-γ and the degranulation activity (CD107^+^) by MC38 tumor-derived T cells on ex vivo stimulation with beads coated with α-CD3/CD28 antibodies. We found that ~10% of CD8^+^ (figure 3F) and ~5% of CD4^+^ (figure 3G) tumor-infiltrating T cells were able to secrete IFN-γ in untreated control mice. Significant increase in the frequency of IFN-γ^+^ CD8^+^ (~20%) and CD4^+^ (~10%) T cells was found in tumors from the hetIL-15-treated group, suggesting that hetIL-15 increases the production of IFN-γ within the tumor. Similarly, we found an increased frequency of degranulating CD107a^+^ CD8^+^ T cells in hetIL-15-treated in comparison to control mice (figure 3F).

Overall, hetIL-15 administration shapes the tumor microenvironment towards a Th1 profile, promoting activation and cytotoxic functional commitment in tumor-resident T and NK cells.

### cDC1 accumulate in tumors of hetIL-15 treated mice

**Figure 4:**
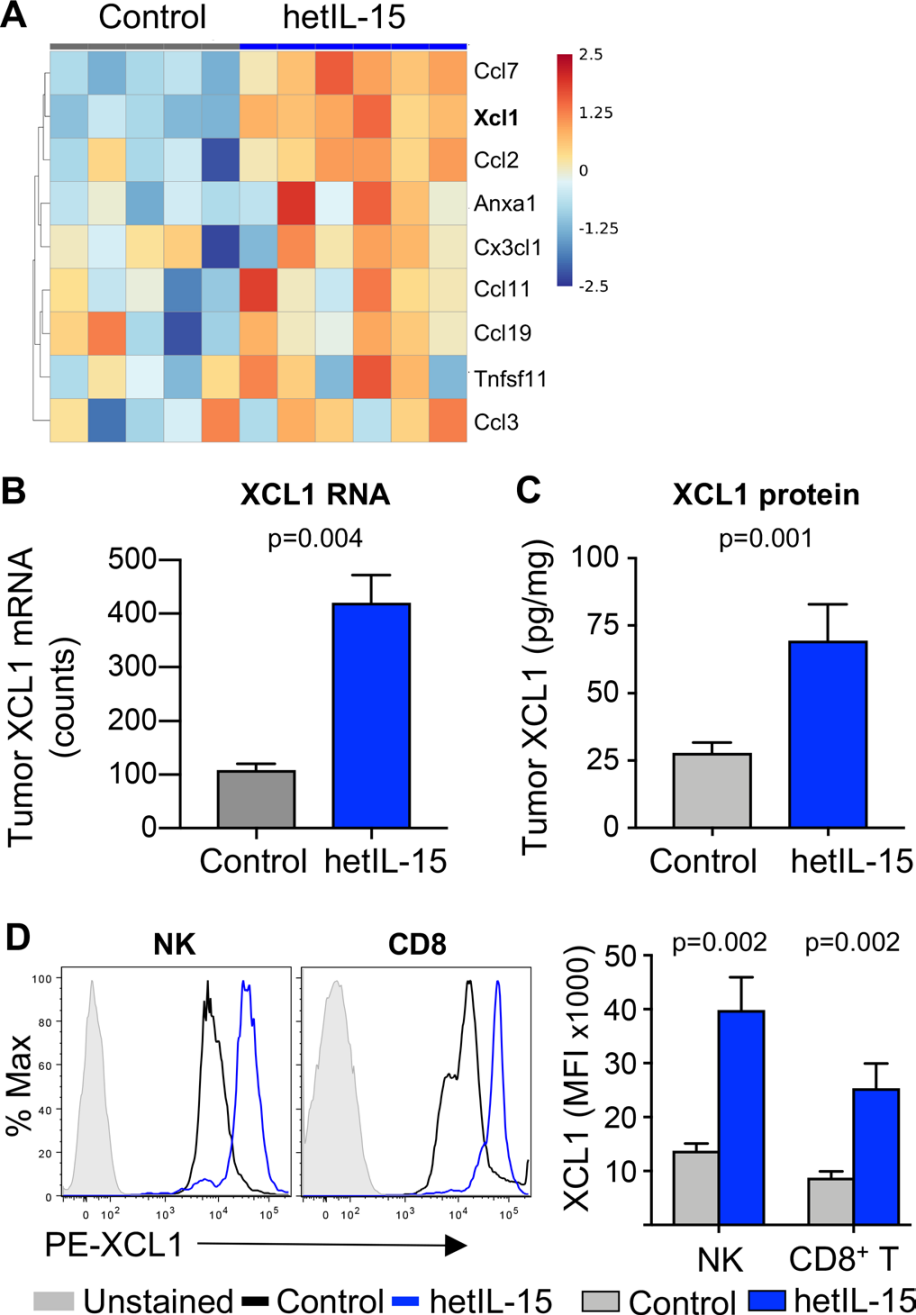
hetIL-15 enhances the production of XCL1 by tumor resident CD8^+^ T and NK cells. (A) Heatmap, represented as Z-score centered and rescaled, of the genes included in the GO:0002548 monocyte migration and chemotaxis pathway from control (n=5) and hetIL-15-treated (n=6) MC38-bearing mice. Significantly upregulated genes in hetIL-15-treated mice in comparison to control mice are depicted. (B) Evaluation of the chemokine XCL1 in MC38 tumors. The panel shows the tumor mRNA counts for *Xcl1* as determined by the Nanostring technologies in both PBS treated (n=5) and hetIL-15-treated (n=6) MC38-bearing mice. (C) MC38 tumor lysates from either PBS-treated (n=11) or hetIL-15-treated (n=10) mice were assessed for XCL1 concentration by ELISA. (D) Flow cytometric analysis of XCL1 production by NK and CD8^+^ T cells. Histogram overlays show the expression of XCL1 by intratumoral NK and CD8^+^ T cells from a representative hetIL-15 (blue) and PBS (black) treated mouse. Solid gray histogram shows non-staining control. The XCL1 geometric mean fluorescent intensity (MFI) in the tumor-infiltrating NK and CD8^+^ T cells from each therapeutic group (n=6) are shown in the right panel. Bars represent mean±SEM. P values are from Mann-Whitney U test. hetIL-15, heterodimeric interleukin-15; NK, natural killer; SEM, Standard error of the mean.

Differential gene expression analysis showed that, in addition to pathways related to NK and T cell activation (online supplementary figure 4), hetIL-15 treatment upregulated several intratumoral chemokines stimulating pathways related to myeloid cell migration and chemotaxis (figure 4A, GO:0002548, p=0.039). Gene expression analysis of the whole tumor revealed a ~4 x increase in the expression of XCL1 mRNA on hetIL-15 administration (figure 4B). Analysis of XCL1 protein within the tumor was performed by ELISA at 6 hours after hetIL-15 IP administration. A ~3 x increase in XCL1 concentration was detected in tumor extracts from hetIL-15 treated mice compared with tumors from PBS treated mice (figure 4C). To identify the source of XCL1, cell suspensions from MC38 tumors were analyzed for the chemokine production in vivo. On day 8 after initiation of treatment, mice received an IT injection of brefeldin to block protein secretion in vivo. Tumors were excised and isolated cells were analyzed by flow cytometry using a panel of antibodies for the detection of NK and T cells as well as XCL1. We found that both tumor-infiltrating NK and CD8^+^ T cells produce XCL1 (figure 4D). hetIL-15 treatment resulted in a significant increase of XCL1 production by both cell subsets, as suggested by increased XCL1 mean fluorescence intensity.

**Figure 5:**
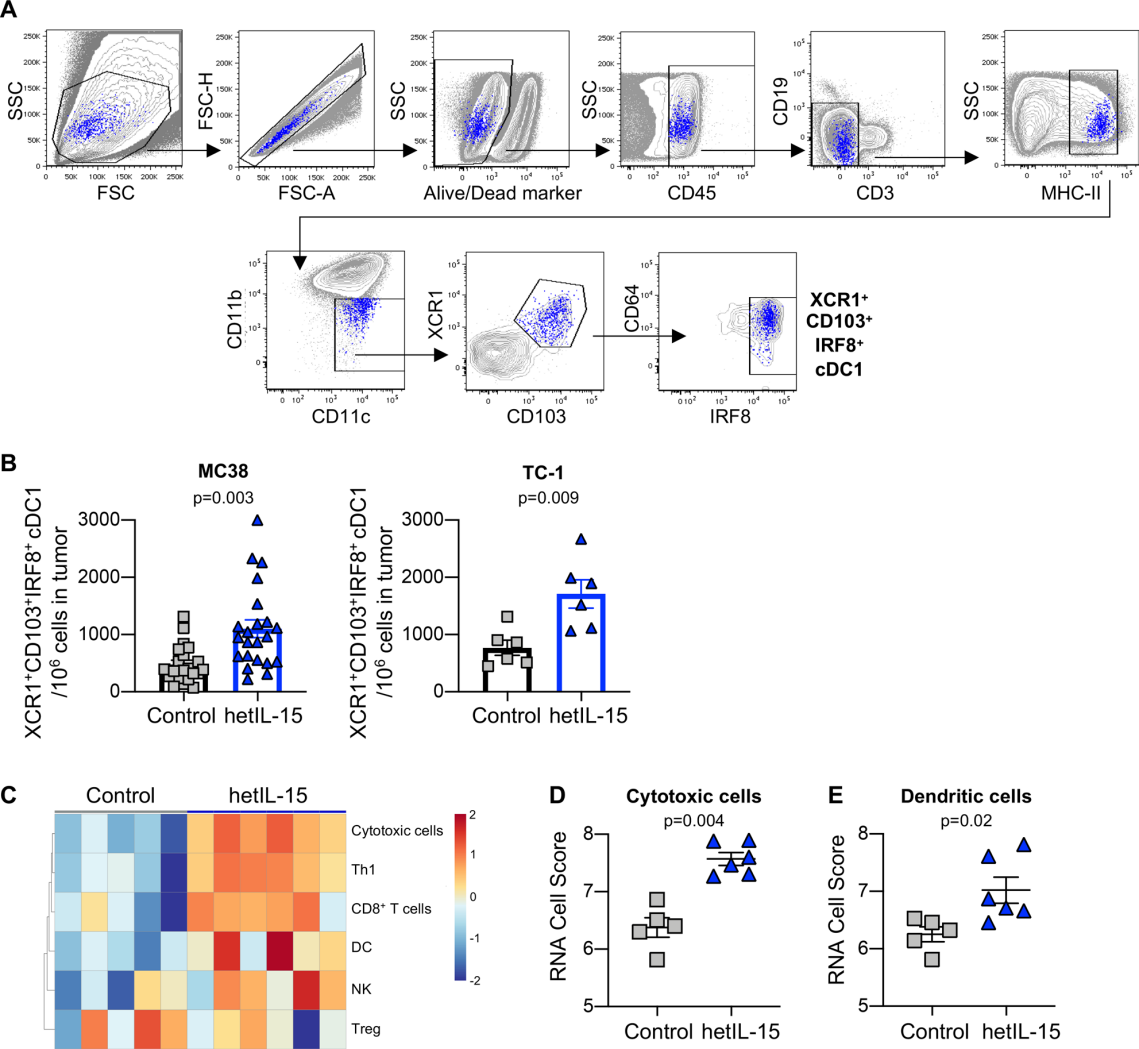
hetIL-15 treatment results in recruitment of XCR1^+^CD103^+^IRF8^+^ cDC1 into tumors. (A) Gating strategy for the characterization of cDC1. (B) The frequency of tumor-infiltrating CD103^+^XCR1^+^IRF8^+^ cDC1 was determined in MC38 (left panel) and TC-1 tumors (right panel) recovered from hetIL-15 or PBS-treated control mice. The number of CD103^+^XCR1^+^IRF8^+^ cDC1 cells in each tumor was normalized per million of cells present in the tumor suspension. The values from individual animals and mean±SEM are shown. (C) Heatmap representing the immune cell composition of tumors on hetIL-15 treatment. Cell scores were calculated for different immune cell subsets as described in material and methods. (D–E) RNA cell score for cytotoxic cells (D) and dendritic cells (E) was calculated for tumors recovered from control (gray, n=5) and hetIL-15 (blue, n=6) treated mice using Nanostring measurements as described in material and methods. Individuals animals and mean±SEM are shown. P values are from Mann-Whitney U test. cDC1, conventional type 1 dendritic cells; hetIL-15, heterodimeric interleukin-15; SEM, Standard error of the mean.

XCL1 has been linked to the tumor recruitment of cDC1.^11 16 55–57^ These cells are known to support antitumor responses through the attraction, stimulation and expansion of tumor-specific CD8^+^ T cells.^58^ For this reason, we evaluated the myeloid cell infiltrate in MC38 tumors on hetIL-15 treatment. A previously established flow cytometry protocol allows the distinction between cDC1, defined as CD45^+^MHCII^high^CD64^-^CD11c^+^CD11b^dim^, and other myeloid populations including CD64^+^ macrophages and CD11b^high^ conventional type 2 DC (cDC2).^13 59^ Additionally, expression of CD103, XCR1 and IRF8 on cDC1 has been linked to their ability to cross-present antigens and stimulate efficient CD8^+^ T cell responses.^14–16 55^ The strategy outlined in figure 5A was used to identify cDC1 expressing XCR1, CD103 and IRF8 within CD45^+^ immune cells infiltrating the tumors. cDC1 constitute approximately 0.1% of intratumoral CD45^+^ leucocytes in MC38 tumors, in agreement with a previous report.^10^ The expression of CD64 on these cells was ~4 x reduced in comparison to MHCII^+^CD11b^+^ myeloid cells (online supplementary figure 5A). Tumor-infiltrating B cells represented between 0.05% and 0.6% of CD45^+^MHCII^+^ and did not stain positive for CD11c, XCR1 and IRF8 (online supplementary figure 5B–D).

Treatment with hetIL-15 resulted in significantly increased accumulation of cDC1 expressing CD103^+^XCR1^+^IRF8^+^ in MC38 tumors (figure 5B, left panel). Expression of the XCL1 receptor (XCR1) on this cell subset suggests its ability to migrate toward the XCL1 gradient within the tumors stimulated by hetIL-15 treatment. Analysis of tumor-infiltrating cDC1 was also performed in control and hetIL-15-treated mice bearing TC-1 carcinoma. Similarly, hetIL-15 treatment was associated with ~2 x increase in the number of cDC1 expressing CD103, XCR1 and IRF8 localized within the tumors (figure 5B, right panel). These results are in agreement with the gene expression data revealed by transcriptomics (Nanostring Technology). Based on the differential expression of cell type specific genes, cell scores were calculated for different immune cell subsets as described in Material and Methods. hetIL-15 treatment induced changes in the composition of the tumor immune infiltrate (figure 5C). hetIL-15 therapy was associated with a significant upregulation of the gene expression profile of both cytotoxic cells (figure 5D) and DCs (figure 5E). In contrast to the results obtained within the tumors, we observed a reduction in the frequency of XCR1^+^CD103^+^IRF8^+^ cDC1 in the spleens of hetIL-15 treated animals compared with control mice (online supplementary figure 3H).

Taken together, these data indicate that hetIL-15 treatment promotes the production of the chemokine XCL1 by intratumoral lymphocytes leading to increased tumor infiltration by CD103^+^XCR1^+^IRF8^+^ cDC1.

### hetIL-15 treatment stimulates CXCL9 and CXCL10 secretion by intratumoral myeloid cells attracting CXCR3^+^ effector T cells by an IFN-γ dependent way

**Figure 6:**
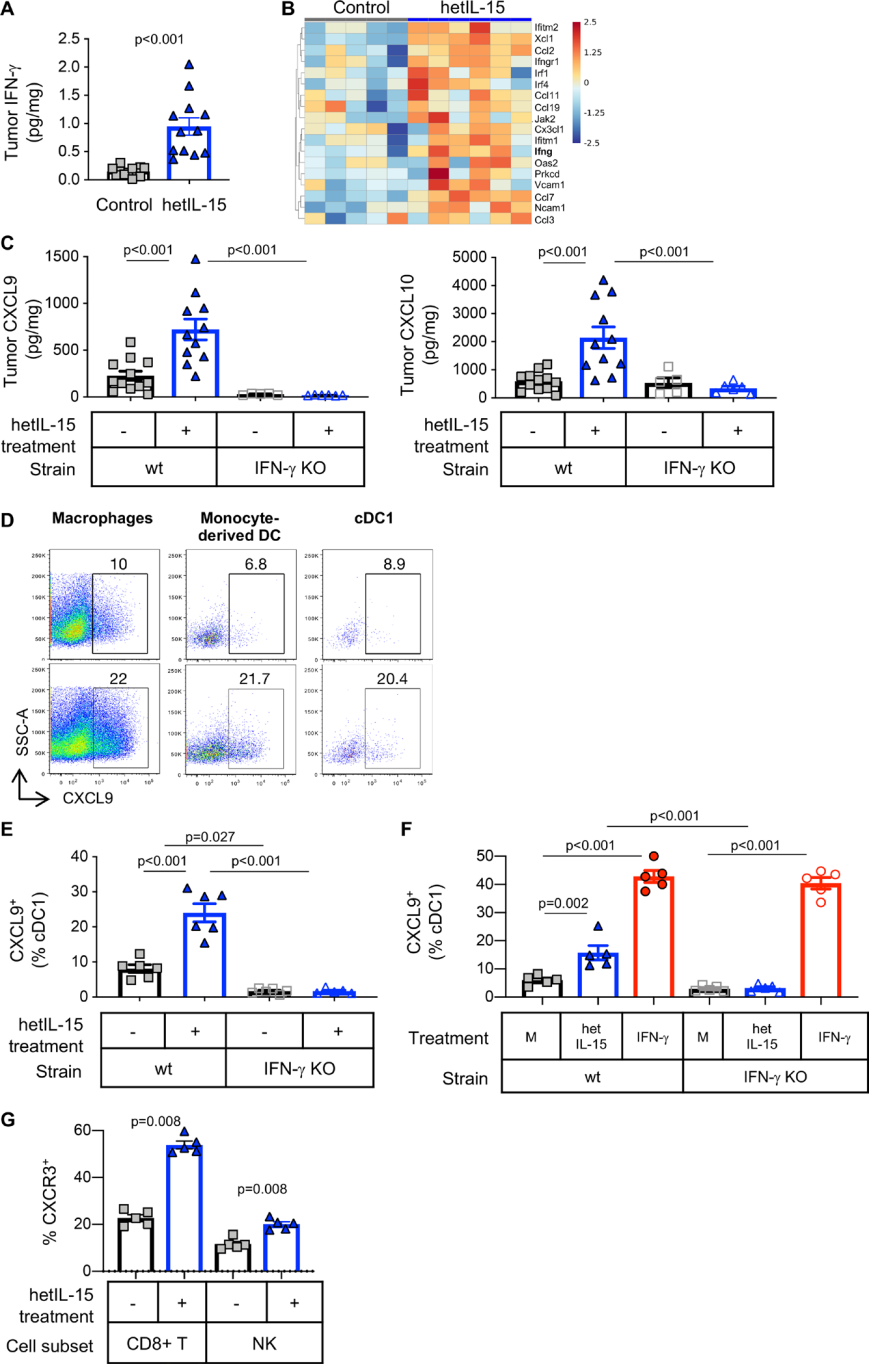
hetIL-15 treatment stimulates secretion of CXCL9 and CXCL10 from tumor-localized myeloid cells in an IFN-γ dependent fashion. (A) Intratumoral IFN-γ concentration measured by U-PLEX MSD in MC38 tumors excised from mice treated with PBS (gray square; n=12) or hetIL-15 (blue triangle; n=12). (B) Heatmap for the genes included in the GO:0034341 IFN-γ related pathway from control (n=5) and hetIL-15-treated (n=6) MC38-bearing mice. Heatmap is represented as Z-score centered and rescaled. Significantly upregulated genes in hetIL-15-treated vs control mice are depicted. (C) Tumor concentration of CXCL9 (left panel) and CXCL10 (right panel) in PBS- (gray squares) or hetIL-15-treated (blue triangles) wt and IFN-γ KO mice. Bars represent mean±SEM. Data from one of two representative experiments are shown. (D) Dot plots showing the in vivo production of CXCL9 by tumor-associated macrophages (defined as MHCII^+^CD64^+^ cells), monocyte-derived dendritic cells (defined as MHCII^+^CD64^-^CD11c^+^CD11b^high^) and cDC1, treated with either PBS (upper panels) or hetIL-15 (lower panels). (E) The frequency of intratumoral cDC1 cells producing CXCL9 is reported as the percentage of the total cDC1 in wt and IFN-γ KO mice treated with PBS (gray square) or hetIL-15 (blue triangle). Bars represent mean±SEM from one representative experiment. (F) CXCL9 production by intratumoral cDC1 recovered from MC38 tumor-bearing wt and IFN-γ KO mice. Cells were stimulated ex vivo with hetIL-15 or IFN-γ and the ability to produce CXCL9 by cDC1 was evaluated by flow cytometry. Bars represent mean±SEM. (G) The frequency of CXCR3^+^ CD8^+^ T cells and NK cells in blood of MC38-bearing mice treated with PBS (gray square) or hetIL-15 (blue triangles) are shown as the percentage of the parental population. Bars represent mean±SEM. P values are from Mann-Whitney U test. cDC1, conventional type 1 dendritic cells; hetIL-15, heterodimeric interleukin-15; IFN-γ, interferon-γ; wt: wild type; KO, knock out; NK, natural killer; SEM, Standard error of the mean.

The intratumoral accumulation of cDC1 and cytotoxic lymphocytes promoted by hetIL-15 was accompanied by increased IFN-γ production by T cells (figure 3) resulting in higher IFN-γ concentration as measured in tumor lysates by Meso Scale Discovery (MSD) quantitation (figure 6A). Differential gene expression analysis showed an increase in IFN-γ mRNA (figure 6B) and IFN-γ responsive genes and confirmed the enrichment of this pathway within the tumor in hetIL-15 treated animals (GO:0034341, p=0.049; figure 6B). The IFN-γ-dependent chemokines CXCL9 and CXCL10 were also assessed in tumor lysates obtained from MC38 bearing-wt and IFN-γ KO mice. Before hetIL-15 treatment, CXCL9 tumor concentration was slightly lower in IFN-γ KO mice compared with wt mice, although this difference did not reach statistical significance by one-way ANOVA (figure 6C, left panel). CXCL10 levels were similar in both strains of mice (figure 6C, right panel). hetIL-15 administration resulted in a ~3 x increase in the intratumoral concentration of both CXCL9 and CXCL10 only in wt mice, with no changes being observed in IFN-γ KO animals (figure 6C). To dissect the mechanism of intratumoral chemokine production in response to hetIL-15, MC38-bearing wt and IFN-γ KO mice were treated with brefeldin delivered IT to block protein secretion in vivo. CXCL9 production in the excised tumors was analyzed by flow cytometry using a mixture of antibodies targeting both T and myeloid cells. We found that all tumor-associated myeloid cell subsets analyzed (macrophages, monocyte-derived DC and cDC1) produce CXCL9 in vivo (figure 6D). hetIL-15 administration increased significantly the frequency of myeloid cells staining positive for CXCL9 in wt mice (figure 6D). cDC1 were one of the sources of CXCL9 (~8% and~2% in wt and IFN-γ KO mice, respectively). The frequency of cDC1 cells producing CXCL9 increased to ~25% in wt mice, but no changes were observed in IFN-γ KO mice (figure 6E), indicating that IL-15-stimulated CXCL9 production is IFN-γ-dependent. To confirm these data, single cell suspensions of MC38 tumors were cultured for 6 hours in the presence of either hetIL-15 or recombinant IFN-γ. Flow cytometry analysis showed that cDC1 produced CXCL9 in response to hetIL-15 only from wt mice(figure 6F). As expected, stimulation with recombinant IFN-γ restored the ability of cDC1 from IFN-γ KO mice to produce CXCL9 to similar levels observed in wt mice (figure 6F). These results are in agreement with the measurements of intratumoral chemokine levels and demonstrate that hetIL-15 stimulation of CXCL9 and CXCL10 production is, at least in part, IFN-γ dependent.

CXCL9 and CXCL10 are key regulators of leucocyte trafficking.^60^ Effector CD8^+^ T cells and a subset of NK cells expresses CXCR3, the receptor for CXCL9, CXCL10 and CXCL11, and they migrate following gradients of these chemokines.^13 23 61^ We observed an increased presence of CD8^+^ T lymphocytes and NK cells expressing CXCR3 in blood of hetIL-15 treated wt mice (figure 6G). Evaluation of CXCR3 expression in TILs was not possible, due to the use of collagenase necessary for tumor processing. Overall, these data support a mechanism of hetIL-15 action in which lymphocyte trafficking to the tumor is controlled by a gradient of the chemokines CXCL9 and CXCL10. Myeloid cells, including macrophages, monocyte-derived DC and cDC1 are among the sources of these chemoattractants in the tumor in an IFN-γ dependent fashion. Hence, the antitumor effects of hetIL-15 treatment are linked to the increase of intratumoral lymphocyte infiltration by an IFN-γ-dependent mechanism.

## Discussion

**Figure 7:**
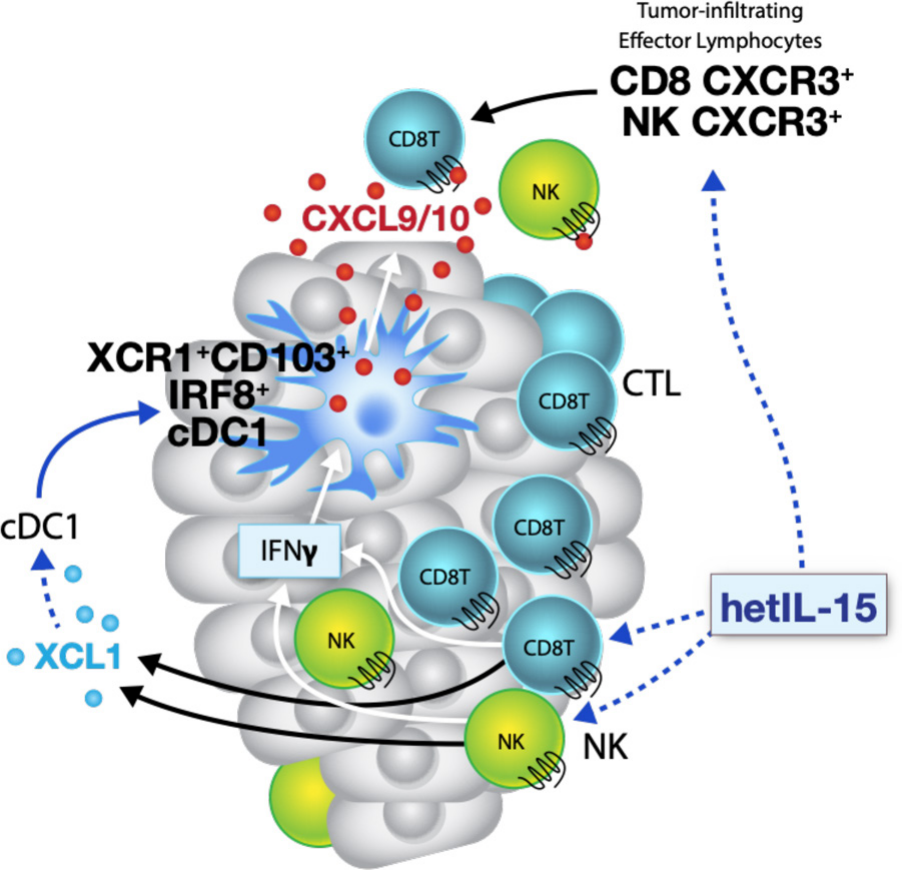
Model representing the hetIL-15 triggered pathway for tumor infiltration by lymphocytes and dendritic cells. Activated CD8^+^ T and NK cells release IFN-γ and XCL1 in response to hetIL-15 treatment. The increased XCL1 concentration recruits cross-presenting cDC1 expressing the chemokine receptor XCR1. cDC1 in tumors respond to hetIL-15 by secreting the chemokines CXCL9 and CXCL10, in an IFN-γ dependent manner. Systemic effects of hetIL-15 includes the enhanced frequency of circulating CXCR3^+^ CD8^+^ T and NK cells that can further infiltrate the tumor, following the CXCL9 and CXCL10 chemokine gradient. cDC1, conventional type 1 dendritic cells; hetIL-15, heterodimeric interleukin-15; IFN-γ, interferon-γ; NK, natural killer.

In this study, using the MC38 colon and TC-1 epithelial carcinoma mouse models, we demonstrate that hetIL-15 orchestrates a multipronged immune response that includes leucocyte expansion and trafficking to the tumor, lymphoid-myeloid cell interactions and enhanced cytotoxic responses through the induction of a favorable cytokine milieu that includes increased intratumoral production of specific chemokines. The increased tumor accumulation of CD8^+^ T and NK cells observed in hetIL-15 treated animals is the result of multiple and concomitant effects, combining both expansion of tumor-resident lymphocytes and recruitment of new immune cells via chemokine gradients. hetIL-15 effects are both direct and indirect and result in control of tumor growth. hetIL-15 directly affects NK, CD8^+^ and CD4^+^ T cells within the tumor promoting their proliferation, survival and cytotoxic commitment, with high levels of IFN-γ production and upregulation of GzmA, GzmB and perforin. Activated lymphocytes also secrete XCL1 in response to hetIL-15 treatment, a chemokine likely responsible for the enhanced intratumoral influx of cDC1 detected in hetIL-15-treated animals. cDC1, together with macrophages and monocyte-derived DC, are one of the sources of the chemokines CXCL9 and CXCL10, previously reported to direct trafficking of CXCR3^+^ effector lymphocytes to tumor sites.^13 23^ Importantly, CXCL9 and CXCL10 secretion by myeloid cells is not constitutive and requires exposure to IFN-γ, suggesting a positive feedback loop whereby the hetIL-15-dependent increase in intratumoral XCL-1 and IFN-γ–producing lymphocytes enhances cDC1 recruitment and amplifies the CXCL9/10-directed intratumoral infiltration by CXCR3^+^ effector lymphocytes, as summarized in the model depicted in figure 7.

The intratumoral leucocyte and cytokine composition orchestrates local interactions that result in either enhancing or inhibiting antitumor immunity. A high number of tumor-infiltrating effector lymphocytes is considered to be the most predictive biomarker for cancer immunotherapy success.^3^ Effective immunotherapeutic agents must promote an immune landscape capable of recruiting T and NK cells within the tumor and enhance their antitumor functions. In our study, tumors had an accelerated growth in IL-15 KO mice, that lack NK cells and have diminished memory CD8^+^ T cells, compared with wt mice. Moreover, administration of hetIL-15 resulted in control of tumor growth in two different cancer models in C57BL/6 mice. hetIL-15 therapy stimulated trafficking of CD8^+^ T and NK cells into the tumors, and promoted proliferation (elevated Ki67 levels) and survival (elevated Bcl-2) of the intratumoral lymphocytes. TILs in hetIL-15-treated tumors were also characterized by a gene expression pattern and phenotype of activated cytotoxic lymphocytes. An important component of the hetIL-15 mediated anti-tumor effects in our study was the increased TILs and CD8^+^/Treg ratio in both MC38 and TC-1 tumors, suggesting that hetIL-15 may be a favorable immunotherapeutic intervention in tumors dependent on Tregs-suppressive functions. Taken together, our data suggest that the recruitment and activity of CD8^+^ T and NK cells within the tumor are responsible for the potent anticancer property of hetIL-15 therapy. These data are in agreement with a recent study demonstrating that endogenous soluble hetIL-15 is expressed in the tumor niche and regulates the frequency of TILs.^25 26^

Using ‘omics’ approaches (Nanostring RNA analysis, and Mesoscale in addition to the ELISA protein measurements), we identified mechanisms responsible for the homing of immune cells to the tumor niche in response to hetIL-15 therapy. hetIL-15 upregulated intratumoral *Xcl1* mRNA expression, and tumor lysates from hetIL-15 treated mice were enriched in XCL1 chemokine. Immunophenotyping analysis of intratumoral lymphocytes revealed activated CD8^+^ T and NK cells as the source of this chemokine, in agreement with previous reports.^11 62 63^ XCL1 increase associated with higher number of CD103^+^XCR1^+^IRF8^+^ cDC1. XCL1 is often cosecreted with IFN-γ, CCL3, CCL4 and CCL5 by CD8^+^ T and NK cells and has emerged as a critical component of the Th1 immune response against several infections.^64^ Dorner *et al* 16 showed that XCL1 is a selective chemoattractant for murine splenic DCs highly efficient in cross presentation. A recent report showed that cDC1 accumulation in transplantable mouse tumors depends on tumor-infiltrating NK cells secreting the chemoattractants CCL5 and XCL1, whereas disruption of this mechanism resulted in cancer immune evasion.^11^ Additionally, in certain human cancers, patient survival correlates with the transcript level of CCL5 and XCL1, which were also positively associated with the abundance of cDC1-like DCs expressing *CLEC9A, XCR1, CLNK* and *BATF3,* highlighting the clinical relevance of the findings. cDC1 uptake dead tumor cells and cross-present tumor-derived peptides to naïve CD8^+^ T cells in the draining lymph nodes, inducing primary cytotoxic responses. Additionally, in this work, we showed that cDC1 contribute also to the recruitment of effector T cells expressing CXCR3, via the secretion of high level of CXCL9, in agreement with a previous finding.^13^

IFN-γ plays a critical role in hetIL-15 mediated antitumor immunity. During hetIL-15 therapy, we observed an upregulation of tumor IFN-γ, both mRNA and protein, together with enhanced expression of the IFN-inducible chemokines CXCL9 and CXCL10. A study by Mikucki *et al* 23 highlighted the mandatory role for the CXCR3/CXCL9/CXCL10 axis in promoting T cell entry into tumors. In agreement with these findings, increased levels of CXCL9 and CXCL10 were reported to be associated with higher density of tumor CD8^+^ T cells, and to correlate with decreased metastatic burden and improved survival in patients with multiple type of cancers.^65 66^ In contrast, CXCL9, CXCL10 and CXCL11 were poorly expressed in ‘cold’ tumors lacking immune infiltrate.^67^ Spranger *et al* 13 showed that the exclusion of CD103^+^ DCs from the tumor microenvironment lead to alteration in the CXCL9/CXCR3 axis and to deficient migration of transferred effector T cells to the tumor, resulting in immunotherapy resistance in non-inflamed tumors. In our study, we reported that cDC1 expressing CD103, XCR1 and IRF8 accumulated at higher density in hetIL-15 treated tumors. Together with other myeloid subsets (macrophages and monocyte-derived DC), CD103^+^XCR1^+^IRF8^+^ cDC1 responded to hetIL-15 therapy by producing CXCL9 and CXCL10 in an IFN-γ dependent fashion. The increased production of IFN-γ by intratumoral lymphocytes could enhance the intratumoral expression of CXCL9 and CXCL10 and subsequent recruitment of the hetIL-15-expanded pool of circulating CXCR3^+^ lymphocytes, generating a positive amplification loop that limits tumor growth.

A comparative analysis between tumor and spleen allowed the identification of hetIL-15 effects restricted to the tumor microenvironement. hetIL-15 resulted in an increased accumulation of GzmB^+^Ki67^-^ CD8^+^ T cells committed to killing preferentially in tumors, supporting the hypothesis that hetIL-15 acts in conjunction with antigen stimulation. Indeed, in a model of adoptive cell transfer, we previously reported that hetIL-15 treatment led to enrichment, in an antigen-dependent manner, of Pmel-1 cells in B16 tumor sites expressing gp100.^47^ Additionally, we observed a reduction in the frequency of splenic XCR1^+^CD103^+^IRF8^+^ cDC1 in hetIL-15 treated animals while their frequency significantly increased in tumors. The mechanism responsible for this difference is currently under investigation. One possibility is their chemokine-driven mobilization towards tumor sites on cytokine treatment.

Preclinical cancer studies support the use of IL-15 to promote the development of antitumor responses by increasing intratumoral lymphocyte infiltration, favoring effector over regulatory cells, and by promoting DC-T cell interactions. Several IL-15 formulations are in different phases of clinical testing in cancer patients. It has been reported that hetIL-15 shows pharmacokinetics and bioactivity superior to single chain monomeric IL-15.^41^ In comparison to single chain IL-15, hetIL-15 has a prolonged serum half-life and is more bioactive on a molar basis.^41^ The superior bioactivity of IL-15 in the heterodimeric formulation is mainly the result of the increased stability of the heterodimeric protein in vivo. These properties allow lower and less frequent dosing, decreasing toxicity. Overall, our results show that hetIL-15 administration, by changing the cytokine and cellular landscape within the tumor, may be a general method to enhance T and cDC1 cell entry in tumors, increasing the success rate of immunotherapeutic interventions.
