# Supplementary material for: Heterodimeric IL-15 delays tumor growth and promotes intratumoral CTL and dendritic cell accumulation by a cytokine network involving XCL1, IFN-γ, CXCL9 and CXCL10
Source: J Immunother Cancer. 2020 May 26;8(1):e000599. doi: 10.1136/jitc-2020-000599 (PMC7254133; doi:10.1136/jitc-2020-000599)
Supplement: Supplementary data [file jitc-2020-000599supp001.pdf]

Supplemental Figure 1

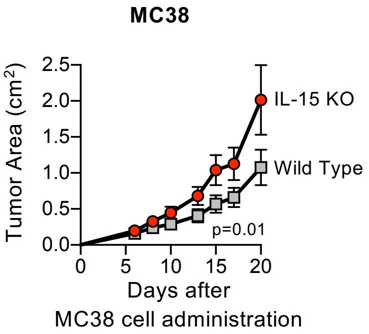

Figure S1. Endogenous IL-15 contributes to tumor control. 10^5 MC38 colon carcinoma cells were administered SC to age-matched wt (grey, n=10) and IL-15 KO (red, n=10) mice. Tumor areas were calculated as length x width and plotted as mean ± SEM for each group overtime.

Supplemental Figure 2

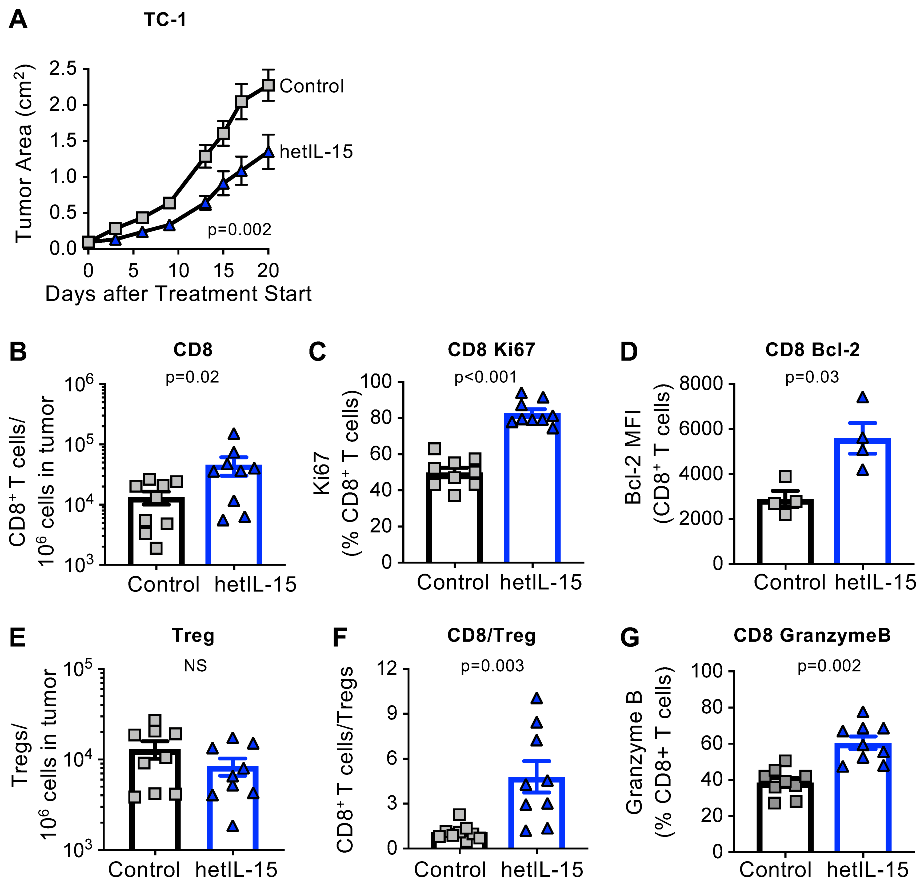

Figure S2. Effects of hetIL-15 on TC-1 carcinomas. (A) Graph showing the TC-1 tumor growth in hetIL-15 treated and control mice. Tumor immune infiltrates from TC-1 carcinomas recovered from mice treated with either PBS or hetIL-15 were analyzed by flow cytometry to determine: (B) the frequency of tumor-infiltrating $CD8^+$ T cells, (C) the percentage of dividing $CD8^+$T cells, (D) the expression of the survival factor Bcl-2 in the tumor-infiltrating $CD8^+$ T cells. The count of tumor-infiltrating Tregs, $CD8^+$ T cells/Treg ratio within the tumor and the percentage of $CD8^+$T cells expressing Granzyme B are shown in (E), (F) and (G) respectively.

Supplemental Figure 3

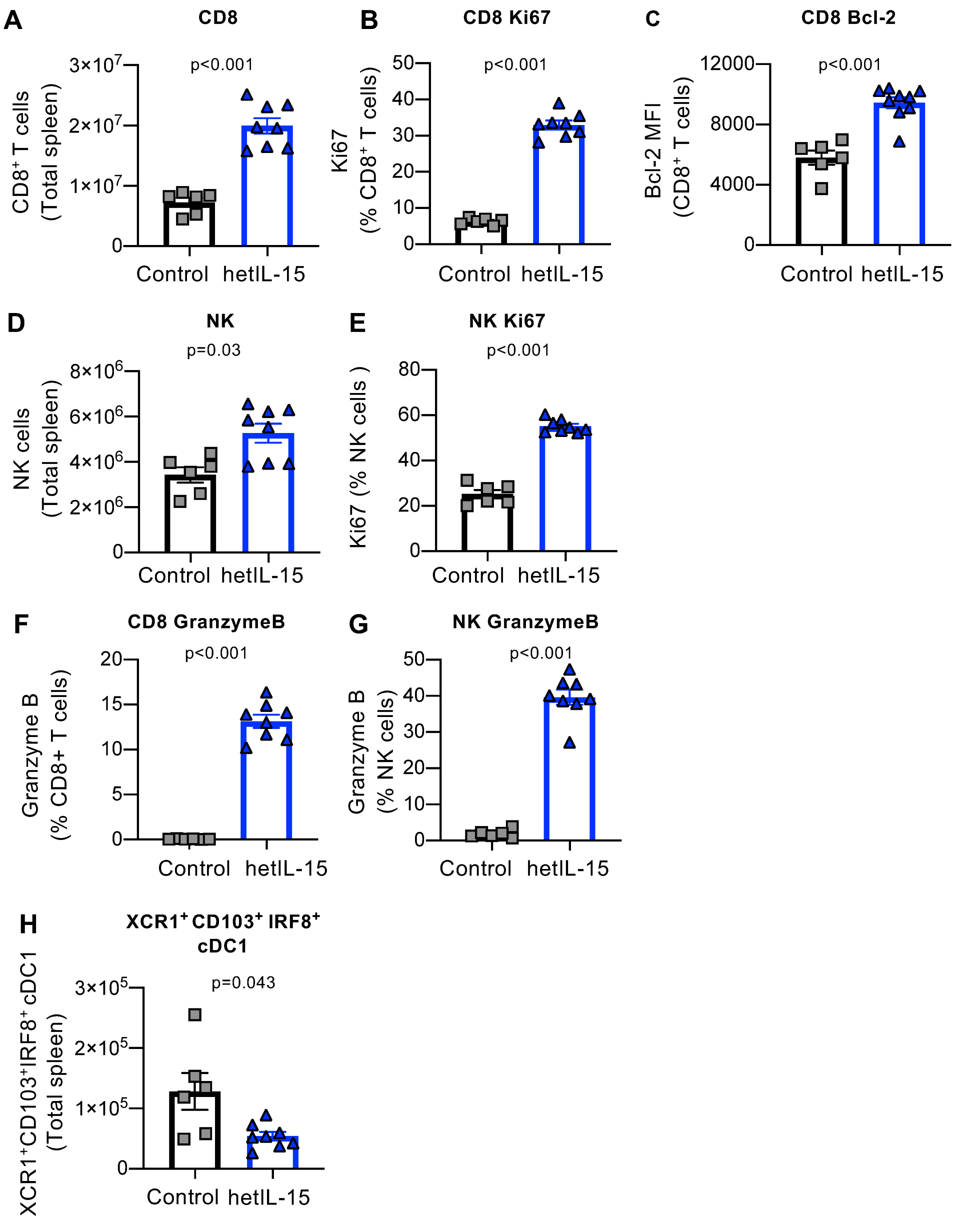

Figure S3. Effects of hetIL-15 in spleens of MC38-bearing mice. Spleen from MC38-bearing mice treated with either PBS or hetIL-15 were analyzed by flow cytometry to determine: (A) the absolute count of $CD8^+$ T cells, (B) the percentage of dividing $CD8^+$T cells, (C) the expression of the survival factor Bcl-2 in the $CD8^+$ T cell subsets, (D) the absolute count of NK cells, (E) the percentage of dividing NK cells, (F) the percentage of $CD8^+$T cells expressing Granzyme B, (G) the percentage of NK cells expressing Granzyme B. Splenic absolute count of $XCR1^+CD103^+IRF8^+$ cDC1 in PBS- and hetIL-15-treated animals is shown in (H).

Supplemental Figure 4

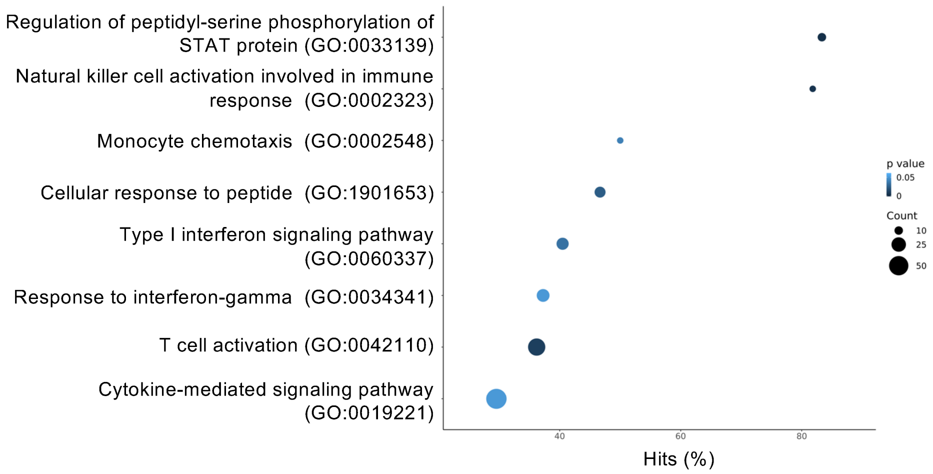

Figure S4. Top GO immune cell-function related pathways significantly enhanced in tumors by hetIL-15 treatment. Nanostring analysis identified up-regulation of genes representing immune-related pathway signatures in tumors by hetIL-15 treatment. Gene enrichment analysis on the GO database was performed on the top 150 analyzed genes (sorted by t-statistic).

Supplemental Figure 5

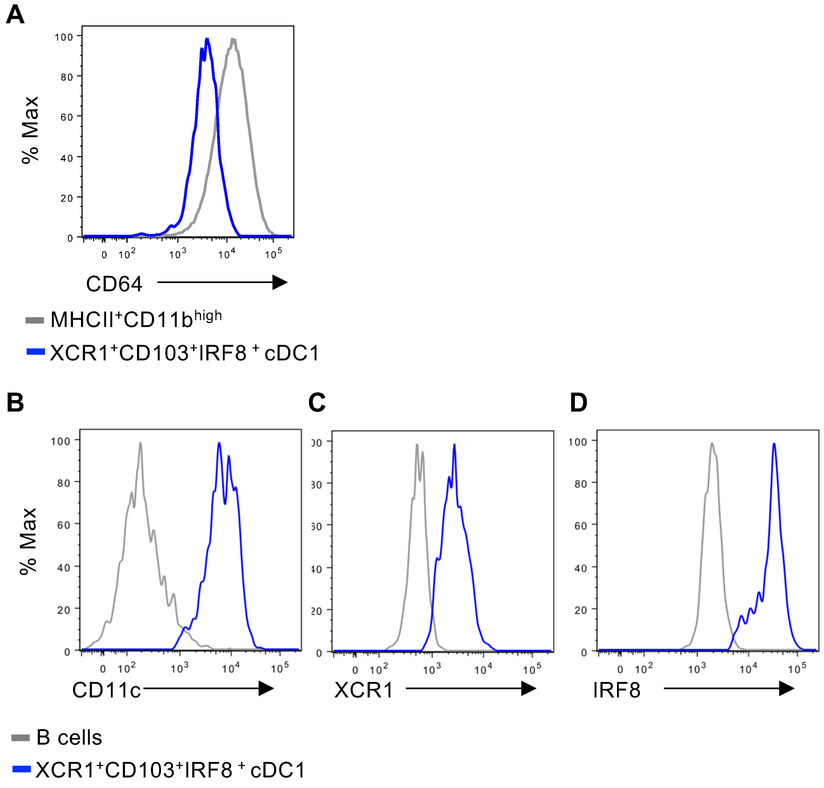

Figure S5. CD11b⁺myeloid and B cells are distinct from cDC1 in tumor. (A) Flow cytometric analysis of CD64 expression by tumor infiltrating MHCII^{+}CD11b^{high} (grey) and XCR1^{+}CD103^{+}IRF8^{+} cDC1 (blue). Expression of CD11c (B), XCR1 (C) and IRF8 (D) by B cells (grey) in comparison to XCR1^{+}CD103^{+}IRF8^{+} cDC1 (blue).
